# A simple and highly sensitive LC–MS workflow for characterization and quantification of ADC cleavable payloads

**DOI:** 10.1038/s41598-024-61522-4

**Published:** 2024-05-14

**Authors:** Shi Ya Mak, Shuwen Chen, Wey Jia Fong, Andre Choo, Ying Swan Ho

**Affiliations:** Bioprocessing Technology Institute (BTI), Agency for Science, Technology and Research (A*STAR), Centros, Singapore, 138668 Singapore

**Keywords:** Biotechnology, Cancer, Drug discovery, Chemistry

## Abstract

Antibody–drug conjugates (ADC) payloads are cleavable drugs that act as the warhead to exert an ADC’s cytotoxic effects on cancer cells intracellularly. A simple and highly sensitive workflow is developed and validated for the simultaneous quantification of six ADC payloads, namely SN-38, MTX, DXd, MMAE, MMAF and Calicheamicin (CM). The workflow consists of a short and simple sample extraction using a methanol-ethanol mixture, followed by a fast liquid chromatography tandem mass spectrometry (LC–MS/MS) analysis. The results showed that well-validated linear response ranges of 0.4–100 nM for SN38, MTX and DXd, 0.04–100 nM for MMAE and MMAF, 0.4–1000 nM for CM were achieved in mouse serum. Recoveries for all six payloads at three different concentrations (low, medium and high) were more than 85%. An ultra-low sample volume of only 5 µL of serum is required due to the high sensitivity of the method. This validated method was successfully applied to a pharmacokinetic study to quantify MMAE in mouse serum samples.

## Introduction

Cancer is one of the leading causes of death worldwide. According to the latest World Health Organization report in 2021, there is an estimation of 19.3 million new cancer cases occurring in 2020^1^. This is a driving force for the rapid emergence of antibody–drug conjugates (ADCs) as a promising class of biotherapeutics for targeted cancer therapy, enabling the selective treatment of cancer cells. To date, fifteen ADCs has been approved by U.S. Food and Drug Administration (FDA), with many more entering clinical trials^2,3^.

Unlike conventional chemotherapy which are non-specific and toxic to normal cells, ADCs use antibodies to deliver cytotoxic drugs to the targeted tumor sites without harming the normal healthy cells in the process. It typically consists of an antibody that can locate and bind to the cancer cell, a linker and a cytotoxic payload that kills the cancer cell^4,5^.

The payload acts as the warhead that exerts an ADC’s cytotoxic effects on cancer cells in the intracellular environment. Ideally, an ADC drug should be stable in blood circulation such that all payloads are delivered to the targeted cancer cells and internalized. However, not all payloads could be successfully delivered to the cancer cells due to premature drug loss in the circulatory system before reaching the targeted cells. This will cause off-target toxicity and side effects in patients^2^.

To evaluate the safety and efficacy of different ADCs, it is important to determine the stability of the ADC molecule. Information on the rate of loss of payloads from its antibody in biological matrices is necessary to assess whether an individual ADC is an effective treatment option. Therefore, analysis of the unconjugated payload is essential in the development of an ADC^6–9^.

With advances in technology, third generation ADCs now have antibodies with improved specificity and lowered immunogenicity. Linkers are also designed to be more stable in blood circulation, releasing drugs only in the targeted cells. These developments have brought about reduced amounts of cytotoxic drugs being administered, thus decreasing the toxicity risk for patients^10–12^. The cytotoxicity activities for various common payloads are in the sub- to low nano nanomolar range as reported by Goldenberg et al.^13^. As a result, to effectively investigate future ADC’s therapeutic window and drug dosage, a sensitive LC–MS/MS method is needed to detect free payloads at similar concentrations for in-vitro and in-vivo studies^6,14,15^. Existing published methods generally quantify in the nanomolar concentration range (Supp. Table S1)^16–19^. Therefore, a highly sensitive LC–MS/MS method as presented in this study would be important in the future development of ADCs.

Furthermore, there is an increasing interest in the conjugation of several classes of payloads onto an individual ADC^3,20–24^. This will enable the ADC to deliver payloads with different mechanism of action of cell-killing, thus increasing its efficiency in destroying cancer cells^3^. To our knowledge, there are no reported methods that quantifies more than one ADC payload in a single LC–MS/MS method. Our LC–MS/MS workflow aims to address this gap by simultaneously characterizing and quantifying six ADC payloads across different classes in a single chromatographic run. This would be of relevance and importance in the future development of ADCs with several payloads.

Herein, we present a highly sensitive and robust workflow validated using the ICH Harmonised guidelines on bioanalytical method validation^25^ to characterize and quantify six unconjugated payloads in serum samples: 7-ethyl-10-hydroxycamptothecin (SN-38), Methotrexate (MTX), Deruxtecan (DXd), Monomethyl auristatin E (MMAE), Monomethyl auristatin F (MMAF) and Calicheamicin (CM). The sample preparation required is simple, with minimal steps and can be completed within 35 min. In addition, the LC–MS analysis is achieved using a short chromatographic run of 11 min and a simple solvent system comprising of methanol, water and formic acid. This workflow is amenable to high-throughput and automated analysis for the screening of free ADC payload in in-vitro and in-vivo studies, to evaluate their stabilities and toxicities in biological sample matrices.

In this study, our workflow was successfully applied to a pharmacokinetic study to quantitate levels of free MMAE in serum obtained from mice after intravenous administration of an ADC.

## Materials and methods

### Chemical and reagents

High purity standards of ADC payloads were purchased from MedChemExpress (New Jersey, USA). Nicotinamide D_4_ obtained from Cambridge Isotope Laboratories Inc. (Massachusetts, USA) was spiked into each sample as internal standard (IS). All standard stocks were reconstituted in LC–MS grade Dimethyl Sulfoxide (DMSO) from Thermo Fisher Scientific (Massachusetts, USA).

Mobile phases prepared for LC analysis were laboratory grade water from a Satorius water purification system (Goettingen, Germany), Optima grade methanol from Fisher Chemical (Pennsylvania, USA) and gradient grade liquid chromatography acetonitrile from Merck (Darmstadt, Germany). In addition, formic acid of ≥ 99%, HiPerSolv CHROMANORM for LC–MS from VWR Chemicals (Pennsylvania, USA) was used as an additive.

Sample preparation was carried out using EMSURE grade ethanol from Merck (Darmstadt, Germany) and Optima grade methanol.

### Biological samples

Method validation was performed using mouse serum from MyBioSource (San Diego, USA, Catalog no.: MBS238204. Lot no.: 155574), which was stored in − 20 °C freezer until use. These were reported as normal mouse serum, 0.2 µm filtered with no preservative added. Furthermore, they are prepared from barrier mice which are screened for infectious agents.

In addition, to further assess the selectivity and matrix effect, serum was obtained from 5 other sources—one rat serum from MyBioSource (San Diego, USA, Catalog no.: MBS238211. Lot no.: 155575), one human serum each from MyBioSource (San Diego, USA, Catalog no.: MBS170604. Lot no.: 11C5346). The remaining human sera were from Sigma Aldrich (St. Louis, Missouri, USA, Catalog no.: H5667. Lot no.: SLCL6524, Catalog no.: H4522 Lot no.: SLCJ 3593 and Catalog no.: H4522, Lot no.: SLCK 9619).

### Preparation of stock solutions and calibrating solutions

Stock solutions of the payload standards were prepared in DMSO at concentrations of 10 mM. These stock solutions were stored in a − 20 °C freezer until use. The calibrating solutions were further prepared by diluting the calibration stock solutions using methanol: ethanol (50% v/v).

### Sample preparation

**Figure 1:**
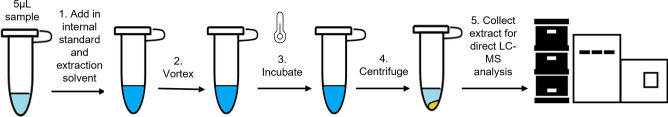
Schematic diagram describing the sample preparation.

A single-phase extraction with methanol-ethanol mixture as the extraction solvent was used to extract the target analytes from the mouse serum (Fig. 1). 5 µL of serum was first spiked with 2 µL of 7.5 µM Nicotinamide-D_4_ as internal standard (IS), then 15 µL of ice-cold methanol: ethanol (50% v/v) was added. The mixture was vortexed for 5 min before leaving it at − 20 °C for 20 min for protein precipitation. Subsequently, the sample was centrifuged for 10 min at 14,000 g, 4 °C. The supernatant was collected and used for LC–MS analysis directly.

### Liquid chromatography (LC) conditions

A Waters Acquity Premier UPLC system (Massachusetts, USA) consisting of a binary pump, thermostatic column holder and a refrigerated sample manager is used. Chromatographic separation is achieved using the Kinetex F5 Core–shell column (2.1 × 100 mm, 1.7 µm, Phenomenex) with 0.1% formic acid in water as mobile phase A and 0.1% formic acid in methanol as mobile phase B. The column temperature and sample manager are maintained at 45 °C and 4 °C respectively. The injection volume was 1 µL. The flow rate was 0.15 mL/min, and the gradient started at 20% B. Over the next 2.0 min, %B was increased to 70% and held for 5.0 min. Finally, the column was flushed at 90% B with a flow rate of 0.3 mL/min for 1.5 min before being equilibrated back to 20% B for 2.5 min. The total run time was 11 min.

### Mass spectrometry conditions

A Waters Xevo TQ-XS triple quadrupole mass spectrometer (Massachusetts, USA) coupled with electrospray ionization interface was used. Samples were analyzed in the positive multiple reactions monitoring (MRM) scan mode. The details on the MRM pairs are described in Table 1.

**Table 1:** MRM acquisition parameters.

Payload (Target analyte)	Precursor (m/z)	Fragment (m/z)	Cone(V)	Collision (eV)
NicotinamideD4	127.1	55.8	40	35
SN38	393.2	249.1	24	46
MTX	455.2	308.1	40	20
DXd	494.2	375.3	60	34
MMAE	718.6	152.0	56	30
MMAF	732.6	170.0	56	34
CM	1368.3	158.1	60	30

### Data analysis

After the LC–MS analysis, data were processed by Waters TargetLynx V4.2 (Massachusetts, USA). Peaks were smoothed using the moving average filter. After smoothing, peaks of each target analyte were detected by its distinctive MRM pair and retention time. For the construction of calibration curves, weighted linear regression models were applied accordingly.

### Pharmacokinetic study of MMAE conjugated ADC in mouse model

- Pharmacokinetic studies in mice model
  A chimeric antibody, CA1, was covalently conjugated with MMAE via a VC linker by disulfide bond reduction to form the ADC. 6 mice (3 female, 3 male) were dosed with the ADC via a single, intravenous tail vein injection at 5 mg/kg. Whole blood sample was collected from tail vein at various timepoints for up to 8 days (0 h, 4 h, 1 day, 2 days, 3 day, 4 days, 7 days, 8 days). The serum fractions (10–20 µL) were then collected after centrifugation and stored at − 80 °C until analysis.
- ELISA quantification of ADC in serum
  96-well plates were coated with 3 µg/mL of MMAE monoclonal antibody (Creative Diagnostics, New York City, USA) overnight at 4 °C. Subsequently, the plates were blocked using 3% BSA/PBS before addition of diluted serum (1000x to 8000x dilution with 1%BSA/PBS) and incubating them for 1 h at 37 °C. The calibration standard ranges from 250 ng/mL to 1.953 ng/mL. Following incubation, the plates were washed three times with 0.05% Tween/PBS and then incubated with anti-IgG-HRP (Sigma-Aldrich, St. Louis, Missouri, USA) at 37 °C for 1 h. OPD (Sigma-Aldrich, St. Louis, Missouri, USA) substrate was subsequently added to wells for development for 1–2 min and quenched using 3M HCl. The plates were analysed for absorbances at 492 nm with 620 nm as reference using a microplate reader (Tecan, Männedorf, Switzerland).
- Computation of pharmacokinetics parameters of ADC and free MMAE
  Pharmacokinetic parameters of free MMAE and ADC were calculated with non-compartmental method using PKSolver Excel add-in program. The maximum free MMAE concentration (C_*max*_) and its corresponding peak time (T_*max*_) can be observed from its serum concentration-time profile. The total exposure of free MMAE, characterized by the area under the curve (AUC_*0-t*_), was calculated by the linear trapezoidal rule.

## Results and discussion

### Method optimization

- LC column chemistry
  Three analytical columns were first evaluated for their ability to separate the individual target analytes. These include the ACQUITY UPLC BEH Phenyl Column (Waters, Massachusetts, USA), ACQUITY UPLC CSH Phenyl-Hexyl Column (Waters, Massachusetts, USA) and Kinetex F5 Core-shell column. These columns have aromatic group stationary phases, featuring favorable levels of π-π interactions that have been reported to provide good retention and selectivity of the analytes, due to the presence of aromatic rings in these compounds.
  Based on the evaluation, it was found that MMAE and MMAF were not well resolved using the BEH Phenyl column (Supp. Figure 1). This separation would be important for development of ADCs with both MMAE and MMAF as payloads. While both the CSH Phenyl-Hexyl and Kinetex F5 Core-shell columns were successful in resolving MMAE and MMAF, the Kinetex F5 Core-shell column was selected for further development, due to increased sensitivity for all analytes, as reflected by an average of 40% increase in integrated peak areas at the same analyte concentrations.
  Although the Phenyl-Hexyl column has a three more carbon propyl linker (trifunctionally bonded C6 phenyl ligands) as compared to the Kinetex F5 column (C3 fluoro-phenyl ligands), this increase in linker hydrophobicity did not help in the selectivity of the analytes. On the other hand, the Kinetex F5 column with a lower hydrophobicity and presence of highly electro-negative fluorine moieties create a rich variety of interaction mechanisms like dipole-dipole, induced dipole and hydrogen bonding^26^ which are beneficial in separating our analytes.
- LC solvent
  Initially, acetonitrile was used as the organic solvent in mobile phase B for chromatographic separation. To further improve the sensitivity of detection and separation of analytes, methanol was also tested. With methanol, the retention time difference between MMAE and MMAF was increased from 0.1 min to 0.19 min. The use of methanol in the mobile phase increased the sensitivities of SN38, MTX, DXd and CM by more than 2-fold. In view of these observations, methanol was chosen over acetonitrile as the solvent for mobile phase B. The improvements in peak resolution and sensitivities using methanol as the mobile phase corroborate the findings from Aqeel et al.^27^, which concluded that methanol encourages π-π interactions of the analytes with the phenyl group stationary phase. In contrast, the π electrons from the nitrile bond in acetonitrile compete for the π-π interactions between phenyl phase and the analytes, leading to poorer analyte retention.
- LC Flow rate and injection volume
  To improve the sustainability of the LC method, additional development was carried out to reduce solvent consumption and sample injection volume needed. The flow rate of 0.3 mL/min with 4 µL injection volume was successfully decreased to 0.15 mL/min with injection volume of 1ul with no compromise in method sensitivity.
- Extraction solvent for sample preparation
  The use of acetonitrile and methanol/ethanol (1:1) as extraction solvents was compared. Analytes extracted using acetonitrile resulted in poor peak shapes when analyzed directly by LC–MS/MS (Supp. Figure 2). This is likely due to the poor solubility of acetonitrile in the mobile phase system consisting of water and methanol. In addition, the need to dry down the extracts and reconstitute in methanol will increase the sample preparation time and result in unnecessary loss of analytes in the process. In consideration of the above factors, methanol/ethanol (1:1) was selected as extraction solvent.

### Method validation

- Sample total recovery
  Extracted samples at low, medium and high concentrations versus extracts of blanks spiked with analyte at the same concentration were determined as shown in Table 2. Recoveries are between 85 and 110%, which showed reproducible and consistent sample preparation at different concentrations.

**Table 2:** Percentage total recovery of different analytes.

Analyte	Spiked concentration (nM)	% Total recovery (n = 3)	
		Mean % ± SD	CV (%)
SN 38	1	92.78 ± 8.78	9.47
	40	98.53 ± 9.78	9.92
	75	105.23 ± 7.41	7.04
MTX	1	88.5 ± 6.93	7.83
	40	93.65 ± 3.41	3.36
	75	100.84 ± 9.96	9.87
DXd	1	92.07 ± 10.12	10.99
	40	97.7 ± 7.08	7.24
	75	98.18 ± 4.66	4.75
MMAE	0.12	105.72 ± 10.48	9.91
	40	103.59 ± 3.76	3.62
	75	94.75 ± 8.87	9.36
MMAF	0.12	105.32 ± 11.66	1.57
	40	104.01 ± 3.74	3.59
	75	94.96 ± 7.47	7.87
CM	1.2	90.52 ± 6.33	6.99
	400	101.94 ± 2.84	2.79
	750	92.2 ± 7.40	8.03
  As described in the materials and method section, the sample preparation is based on a single-phase extraction. It is a straightforward method whereby the extracted samples can be injected into the mass spectrometer directly. With only simple aliquoting and dispensing of liquids involved in preparation, this method can be easily automated for large scale analysis. In addition, the low sample volume needed (5 µL) would be advantageous in conditions whereby the sample volume is a limiting factor.
- Selectivity
  Six individual serum sources (1 mouse, 4 human and 1 rat) were evaluated for selectivity. No peaks of interference were observed at the retention times of analytes and IS in all serum sources. Figure 2 shows the representative chromatograms of an extracted blank serum and the peaks observed for respective analyte standards.

**Figure 2:**
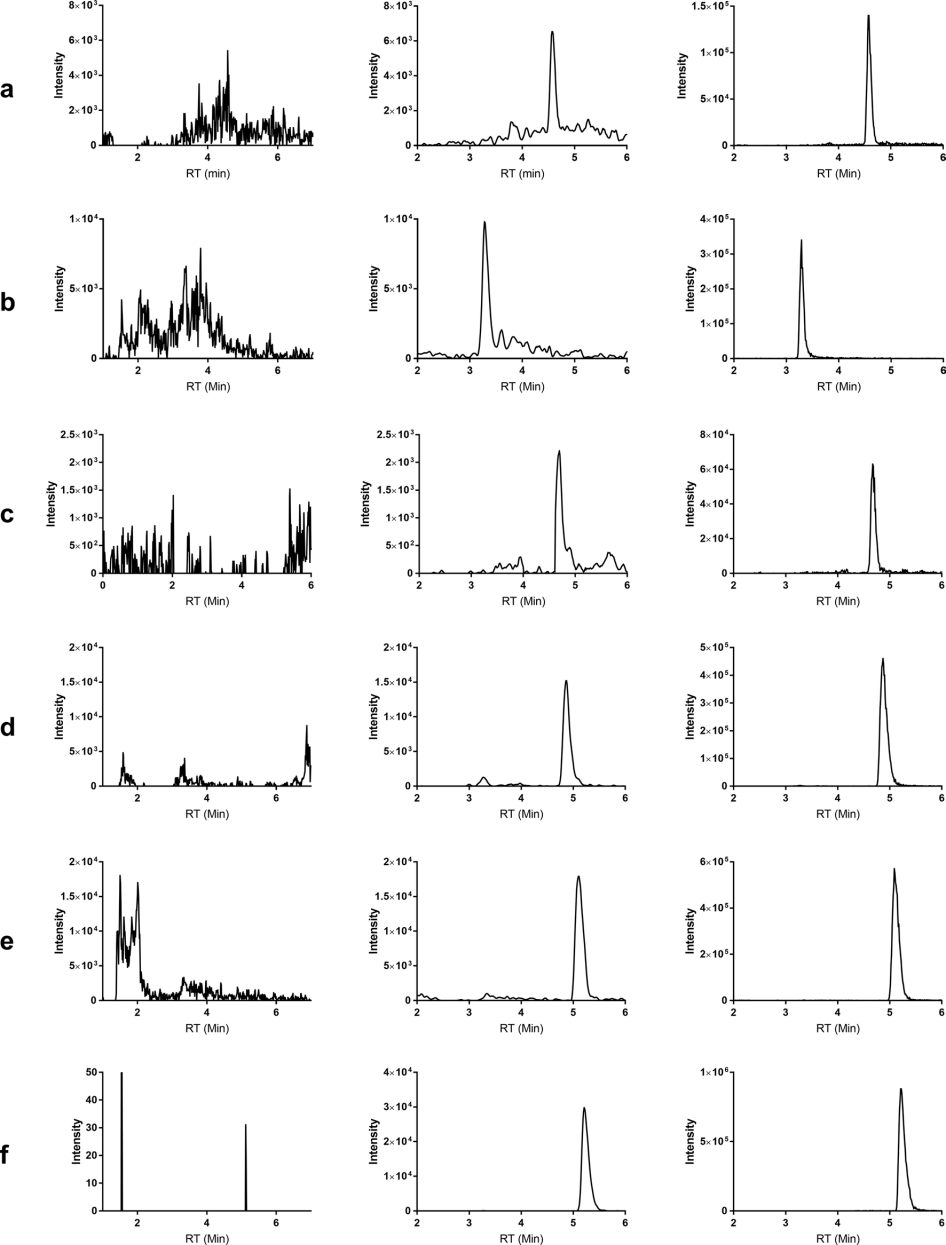
Representative chromatogram of blank serum extract (left column), standards spiked in serum extract at LLOQ (middle column), and standards spiked in serum extract at 10 nM (right column): (**a**) SN38; (**b**) MTX; (**c**) DXd; (**d**) MMAE; (**e**) MMAF; (**f**) CM.

### Calibration curve and linear range

**Figure 3:**
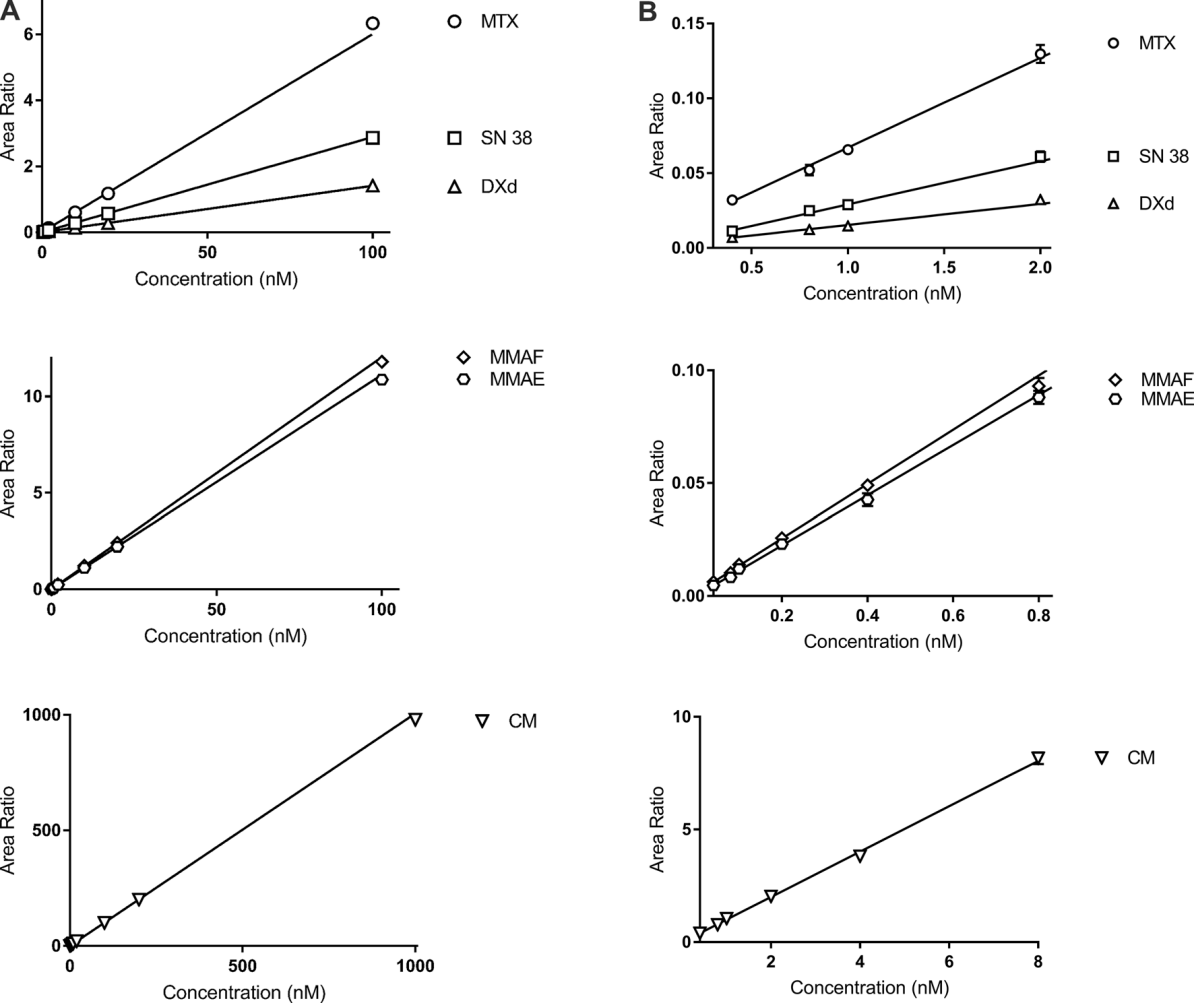
(**a**) Full calibration curves of different analytes; (**b**) Lower range of calibration curves for different analytes.

We added all calibrators in blank mouse serum matrix. Using our optimized extraction method and LC–MS conditions, 3 technical replicates for each calibration point were performed. The CV of our technical replicates were below 20%. Area ratios were calculated using the area of each analyte at each concentration against the area of internal standard. Using a weighted linear regression model of 1/x^2^ factor, we achieved a well-validated linear range of 0.4–100 nM for SN38, MTX and DXd, 0.04–100 nM for MMAE and MMAF, 0.4–1000 nM for CM. LLOQ was within 20% deviation from theoretical concentration while the remaining calibrators were well within 15% deviation (Table 3 and Figure 3).

**Table 3:** Calibration data for different analytes.

Analyte	Concentration of calibration standards (nM)	Within runs (n = 3)			Between runs (n = 3)		
		Back calculated concentration (mean ± SD)	Precision (%) RSD	Accuracy (%) RE	back calculated concentration (mean ± SD)	Precision (%) RSD	Accuracy (%) RE
SN 38	0.4	0.39 ± 0.04	8.56	104.94	0.40 ± 0.04	9.02	100.94
	0.8	0.85 ± 0.04	2.99	105.71	0.83 ± 0.07	8.05	104.17
	1	0.98 ± 0.06	7.41	98.47	1.00 ± 0.08	7.70	99.69
	2	2.06 ± 0.12	6.53	102.84	2.05 ± 0.19	9.21	102.54
	10	9.29 ± 0.62	4.76	92.92	9.82 ± 0.61	6.16	98.18
	20	19.14 ± 0.54	2.79	95.71	19.28 ± 0.85	4.39	96.42
	100	96.67 ± 1.20	1.96	96.67	96.75 ± 8.08	8.35	96.75
MTX	0.4	0.40 ± 0.05	7.99	98.77	0.41 ± 0.04	10.24	101.32
	0.8	0.74 ± 0.06	4.62	92.30	0.77 ± 0.06	7.62	96.43
	1	0.97 ± 0.04	3.08	97.09	0.99 ± 0.05	4.62	99.46
	2	2.04 ± 0.10	7.69	101.81	2.02 ± 0.14	6.69	101.09
	10	10.02 ± 0.27	0.34	100.20	10.05 ± 0.34	3.36	100.54
	20	19.60 ± 0.28	1.75	97.98	20.11 ± 0.55	2.73	100.57
	100	105.97 ± 1.40	1.85	105.97	101.23 ± 9.62	9.51	101.23
DXd	0.4	0.40 ± 0.03	5.50	98.77	0.40 ± 0.03	6.72	100.58
	0.8	0.80 ± 0.09	11.44	99.81	0.80 ± 0.07	9.04	100.53
	1	0.96 ± 0.10	8.66	95.77	0.95 ± 0.06	6.06	95.42
	2	1.94 ± 0.11	4.67	96.80	2.09 ± 0.15	7.27	104.44
	10	9.87 ± 1.07	9.39	98.66	9.97 ± 0.71	7.17	99.71
	20	19.72 ± 1.05	4.27	98.62	19.92 ± 0.68	3.42	99.62
	100	100.15 ± 3.60	4.30	100.15	98.58 ± 8.22	8.34	98.58
MMAE	0.04	0.04 ± 0.00	7.85	95.89	0.04 ± 0.00	8.83	98.42
	0.08	0.08 ± 0.01	10.85	100.47	0.08 ± 0.01	9.57	99.40
	0.1	0.10 ± 0.01	10.91	103.89	0.11 ± 0.01	9.47	105.94
	0.2	0.21 ± 0.01	2.79	103.24	0.20 ± 0.02	8.30	102.03
	0.4	0.39 ± 0.02	4.61	96.53	0.40 ± 0.02	4.28	100.36
	0.8	0.78 ± 0.02	1.53	97.59	0.80 ± 0.03	3.49	100.15
	1	0.97 ± 0.04	3.40	97.18	0.98 ± 0.04	4.57	97.77
	2	2.07 ± 0.21	9.38	103.61	2.03 ± 0.14	6.95	101.69
	10	9.82 ± 0.13	1.88	98.15	9.99 ± 0.24	2.40	99.86
	20	19.67 ± 0.18	0.73	98.35	19.76 ± 0.27	1.37	98.81
	100	98.40 ± 0.98	0.98	98.40	98.21 ± 9.98	10.16	98.21
MMAF	0.04	0.04 ± 0.00	6.18	101.39	0.04 ± 0.00	10.64	100.31
	0.08	0.07 ± 0.00	7.80	92.00	0.07 ± 0.01	7.67	93.24
	0.1	0.10 ± 0.01	10.60	104.77	0.11 ± 0.01	6.71	106.62
	0.2	0.19 ± 0.02	8.81	96.75	0.20 ± 0.02	8.29	101.49
	0.4	0.40 ± 0.02	3.39	100.61	0.40 ± 0.02	4.16	100.39
	0.8	0.79 ± 0.01	2.28	98.52	0.80 ± 0.04	4.82	99.48
	1	0.98 ± 0.04	3.41	98.17	0.98 ± 0.05	4.71	97.79
	2	2.09 ± 0.08	3.04	104.43	2.07 ± 0.12	6.03	103.44
	10	9.92 ± 0.12	0.73	99.22	10.02 ± 0.22	2.23	100.16
	20	19.96 ± 0.34	0.85	99.79	19.86 ± 0.29	1.48	99.31
	100	98.19 ± 1.61	0.95	98.19	98.42 ± 9.05	9.19	98.42
CM	0.4	0.40 ± 0.05	11.67	99.29	0.39 ± 0.04	11.04	98.38
	0.8	0.75 ± 0.05	8.68	94.35	0.80 ± 0.07	8.77	100.38
	1	1.03 ± 0.10	9.20	103.24	1.05 ± 0.08	7.52	105.36
	2	2.03 ± 0.13	4.81	101.55	2.02 ± 0.14	6.90	101.22
	4	3.88 ± 0.05	2.58	97.01	3.93 ± 0.22	5.59	98.28
	8	7.81 ± 0.16	1.50	97.57	8.06 ± 0.32	3.99	100.71
	10	10.26 ± 0.06	0.96	102.63	10.09 ± 0.61	6.06	100.92
	20	20.56 ± 0.34	0.85	102.80	20.26 ± 0.87	4.31	101.30
	100	100.41 ± 1.78	1.25	100.41	102.58 ± 4.91	4.79	102.58
	200	195.41 ± 2.99	0.88	97.71	191.79 ± 9.95	5.19	95.90
	1000	978.70 ± 12.68	1.56	97.87	997.00 ± 72.32	7.25	99.70

- Sensitivity
  Sensitivity is determined by conducting 5 replicate injections in 3 independent runs. All analyte response at LLOQ are more than 5 times of the analyte response of the zero calibrator^28^. The accuracy is less than ± 20% of spiked concentration and precision is less than ± 20% CV, which are compliant with the ICH Harmonised guidelines. These are shown in Table 4.

**Table 4:** Accuracy and precision data for different analytes at low, medium and high QC concentrations.

Analyte	Spiked concentration (nM)	Within runs (n = 5)			Between runs (n = 3)		
		Measured (mean ± SD)	Precision (%)	Accuracy (%)	Measured (mean ± SD)	Precision (%)	Accuracy (%)
SN 38	0.4	0.42 ± 0.03	10.40	104.94	0.04 ± 0.03	8.29	101.13
	1	1.06 ± 0.06	6.58	105.56	1.04 ± 0.07	6.37	103.83
	40	42.31 ± 1.57	1.89	105.78	40.87 ± 1.53	3.74	102.18
	75	83.06 ± 1.69	2.26	110.75	78.04 ± 4.47	5.72	104.05
MTX	0.4	0.41 ± 0.03	7.99	102.80	0.42 ± 0.04	10.08	105.47
	1	0.96 ± 0.06	6.93	95.58	0.97 ± 0.05	5.57	97.43
	40	35.58 ± 0.71	0.32	88.96	36.95 ± 1.98	5.34	92.38
	75	71.00 ± 1.17	1.63	94.66	74.66 ± 3.11	4.16	99.54
DXd	0.4	0.41 ± 0.06	9.62	101.85	0.40 ± 0.04	9.46	100.29
	1	1.12 ± 0.13	9.81	111.56	1.04 ± 0.15	14.79	104.30
	40	44.15 ± 1.33	2.65	110.38	43.25 ± 1.24	2.86	108.13
	75	82.83 ± 2.06	2.04	110.44	81.75 ± 3.99	4.88	108.99
MMAE	0.04	0.04 ± 0.01	11.77	100.93	0.40 ± 0.00	11.22	99.29
	0.12	0.13 ± 0.01	10.52	110.37	0.13 ± 0.02	12.19	104.52
	40	36.85 ± 0.71	1.64	92.12	38.79 ± 1.70	4.38	96.98
	75	79.91 ± 1.92	1.19	106.55	77.58 ± 2.72	3.51	103.45
MMAF	0.04	0.04 ± 0.01	11.52	96.94	0.04 ± 0.00	12.06	99.68
	0.12	0.11 ± 0.01	10.33	92.27	0.12 ± 0.02	12.13	103.11
	40	37.67 ± 0.80	1.33	94.18	39.14 ± 1.42	3.63	97.86
	75	77.89 ± 1.67	1.36	103.85	76.31 ± 1.85	2.42	101.75
CM	0.4	0.39 ± 0.08	13.85	97.25	0.40 ± 0.05	13.81	99.00
	1.2	1.28 ± 0.13	10.36	106.40	1.19 ± 0.15	12.40	99.32
	400	369.78 ± 10.33	1.11	92.44	391.50 ± 20.98	5.36	97.87
	750	764.58 ± 22.54	1.05	101.94	758.97 ± 20.97	2.76	101.20
- Accuracy and precision
  Accuracy and precision were likewise studied by performing 5 replicate injections in 3 independent runs Four concentration levels within the linear range of the calibration curves were tested: the LLOQ, within three times of LLOQ (low QC), around 30-40% of the calibration curve range (medium QC) and at least 75% of the ULOQ (high QC). These are shown together in Table 4.
- Matrix effect
  Matrix effect is further evaluated by analyzing 3 replicates of low and high QCs, each prepared using matrix from another 5 different sources in accordance to ICH guidelines. The accuracy is within 15% of the nominal concentration and the precision is not greater than 15%. The results are presented in Table 5.

**Table 5:** Matrix effect assessment for each analyte in different serum matrices.

Matrix source	Analyte	Spiked concentration (nM)	Replicates (n = 3)		
			measured (mean ± SD)	Precision (%)	Accuracy (%)
Human Serum from MyBioSource Catalog no.: MBS170604 Lot no.: 11C5346	SN 38	1	1.00 ± 0.08	7.81	99.64
		75	75.55 ± 3.54	4.68	100.74
	MTX	1	0.93 ± 0.03	3.08	92.64
		75	74.37 ± 2.45	3.29	99.16
	DXd	1	1.02 ± 0.13	12.81	101.77
		75	78.83 ± 3.46	4.38	105.11
	MMAE	0.12	0.12 ± 0.00	2.27	102.10
		75	70.38 ± 2.27	3.22	93.84
	MMAF	0.12	0.13 ± 0.00	0.55	112.03
		75	73.43 ± 3.09	4.21	97.91
	CM	1.2	1.35 ± 0.12	9.17	112.81
		750	768.63 ± 23.52	3.06	102.48
Human Serum from Sigma Aldrich Catalog no.: H4522 Lot no.: SLCJ 3593	SN 38	1	0.96 ± 0.08	8.80	96.47
		75	73.73 ± 5.10	6.20	98.31
	MTX	1	0.97 ± 0.03	2.92	97.37
		75	77.00 ± 3.39	4.40	102.66
	DXd	1	1.05 ± 0.06	5.48	105.07
		75	74.58 ± 4.72	6.33	99.44
	MMAE	0.12	0.11 ± 0.01	9.84	95.51
		75	64.51 ± 3.34	5.18	86.01
	MMAF	0.12	0.12 ± 0.01	6.44	96.12
		75	71.77 ± 2.63	3.66	95.70
	CM	1.2	1.12 ± 0.05	4.66	93.68
		750	705.67 ± 49.02	6.95	94.09
Human Serum from Sigma Aldrich Catalog no.: H4522 Lot no.: SLCK 9619	SN 38	1	0.97 ± 0.03	3.54	96.97
		75	71.18 ± 1.07	1.51	94.91
	MTX	1	1.04 ± 0.04	4.02	104.01
		75	77.07 ± 3.39	4.40	102.75
	DXd	1	1.03 ± 0.11	10.97	102.73
		75	73.76 ± 2.49	3.37	98.35
	MMAE	0.12	0.10 ± 0.01	11.63	85.37
		75	74.82 ± 2.58	3.45	99.76
	MMAF	0.12	0.11 ± 0.00	3.86	87.64
		75	73.94 ± 2.20	2.98	98.59
	CM	1.2	1.10 ± 0.13	11.46	91.81
		750	713.92 ± 16.22	2.27	95.19
Human Serum from Sigma Aldrich Catalog no.: H5667 Lot no.: SLCL6524	SN 38	1	1.02 ± 0.12	11.60	102.24
		75	81.14 ± 4.14	5.10	108.19
	MTX	1	0.87 ± 0.03	3.14	87.24
		75	72.67 ± 3.63	5.00	96.89
	DXd	1	1.06 ± 0.08	7.38	105.95
		75	81.77 ± 2.80	3.42	109.02
	MMAE	0.12	0.13 ± 0.01	5.29	108.46
		75	68.82 ± 3.27	4.75	91.76
	MMAF	0.12	0.13 ± 0.00	3.28	111.14
		75	71.19 ± 3.30	4.64	94.91
	CM	1.2	1.34 ± 0.04	3.19	111.37
		750	824.77 ± 39.62	4.80	109.97
Rat Serum from MyBioSource Catalog no.: MBS238211 Lot no.: 155575	SN 38	1	1.09 ± 0.06	5.28	108.78
		75	80.45 ± 1.27	1.58	107.27
	MTX	1	1.08 ± 0.04	3.83	108.21
		75	71.75 ± 1.76	2.45	95.67
	DXd	1	1.05 ± 0.13	12.61	104.65
		75	78.99 ± 3.70	4.68	105.33
	MMAE	0.12	0.12 ± 0.01	10.41	103.84
		75	73.54 ± 0.83	1.13	98.05
	MMAF	0.12	0.12 ± 0.00	1.64	100.28
		75	73.32 ± 1.30	1.77	97.76
	CM	1.2	1.30 ± 0.09	6.56	108.74
		750	727.86 ± 6.62	0.91	97.05

### Pharmacokinetics study of MMAE conjugated ADC in mouse model

**Figure 4:**
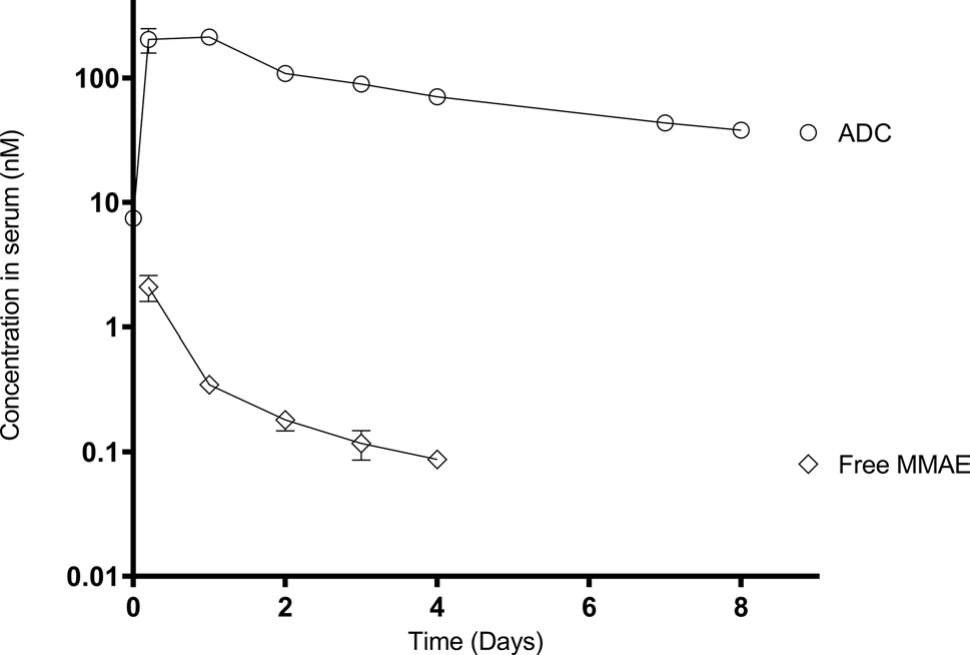
ADC and free MMAE concentration vs time pK profile.

The validated LC–MS method was successfully applied to the pharmacokinetic study to quantitate levels of free MMAE in mice after intravenous administration of ADC at 5 mg/kg. Endogenous level of MMAE was not detected in mouse serum collected before administration. The serum concentration—time profile of ADC and free MMAE (n = 6) is shown in Fig. 4.

Following administration of ADC in mice, free MMAE was released from the ADC due to antibody degradation and toxin deconjugation. However, very low levels of free MMAE (C_max_ = 2.10 ± 0.50 nM), which is about 1% concentration of ADC in serum, were detected in circulation and this suggested the stability of the linker and limited deconjugation. This observation was consistent with the MMAE conjugated anti-EGFR pK study done by Hu et al.^29^ although they had used a higher dose of 15 mg/kg subcutaneously. The reported t_1/2_ value of 38 h by Hu. et al. in their study was also similar to our observed t_1/2_ value of 43 h. Our AUC_0-t_ value of 2.03 ± 0.37 nmol.d/L was also dose proportional to their reported value.

## Conclusion

We have established a reliable and robust LC–MS workflow which was validated according to the ICH guidelines. A combination of the unique properties of the Kinetex F5 column stationary phase, methanol intrinsic solvent characteristic which supports π–π interactions and reduction in flow rate, resulted in a high sensitivity of 400 picomolar and below for the quantification of 6 well-established payloads achieved in a single chromatographic method simultaneously. With the simple sample preparation protocol and fast LC–MS/MS analysis, the entire workflow could be completed within 50 min. This method could also be integrated into an automated workflow for high throughput analysis. While current ADCs comprise of a single payload, there is a potential to engineer payloads of different drug classes onto the antibody for better cancer cell killing efficiency. As such, having a single method for the quantification of two or more payloads would prove to be of relevance for future ADC development.

### Supplementary Information

Supplementary Information.

## Data Availability

The datasets used and/or analysed during the current study available from the corresponding author on reasonable request.
