# Supplementary material for: A simple and highly sensitive LC–MS workflow for characterization and quantification of ADC cleavable payloads
Source: Sci Rep. 2024 May 14;14:11018. doi: 10.1038/s41598-024-61522-4 (PMC11094190; doi:10.1038/s41598-024-61522-4)
Supplement: Supplementary file 1 — Supplementary Information. [file 41598_2024_61522_MOESM1_ESM.docx]

**Supplementary Figures**


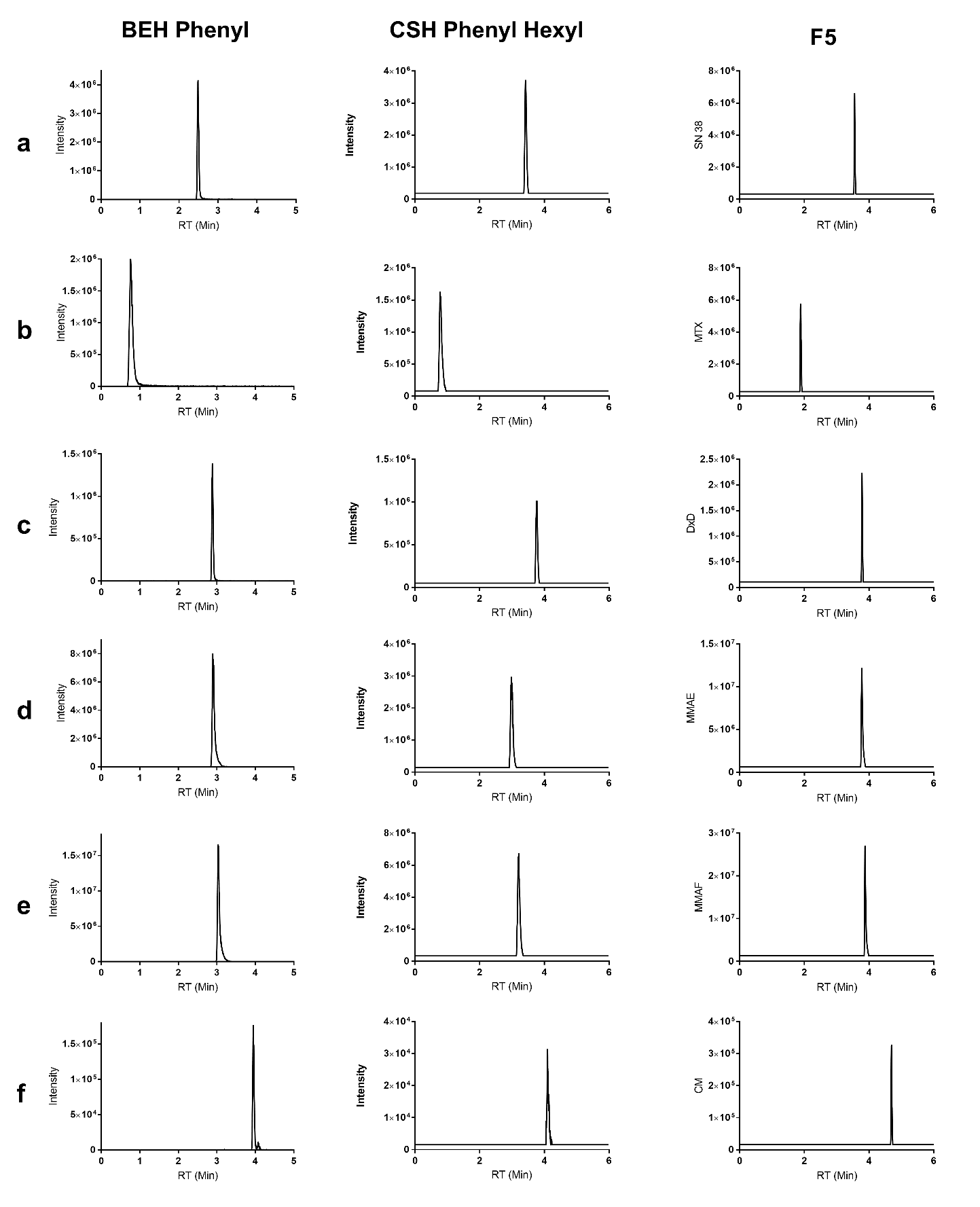


Supplementary Figure 1. Representative chromatogram of standards ran on BEH Phenyl, CSH Phenyl Hexyl and F5 Column: (a) SN38; (b) MTX; (c) DXd; (d) MMAE; (e) MMAF; (f) CM


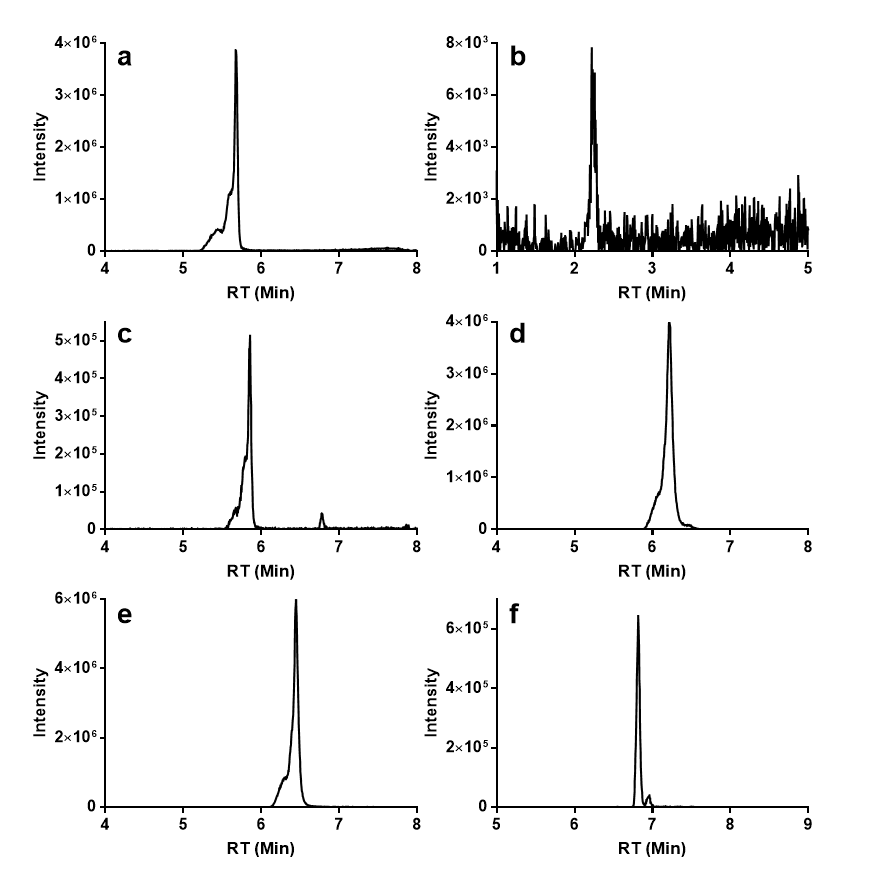

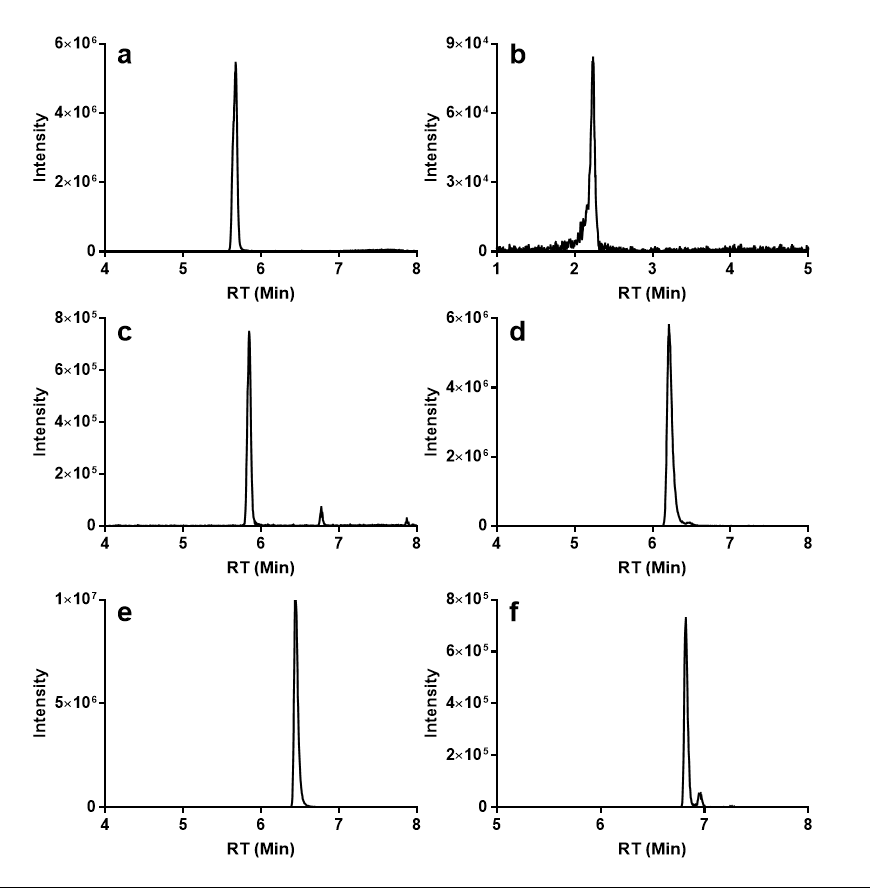


Supplementary Figure 2. Representative chromatogram of standards ran using acetonitrile as extraction solvent (left) and Ethanol: Methanol as extraction solvent (right): (a) SN38; (b) MTX; (c) DXd; (d) MMAE; (e) MMAF; (f) CM

Supplementary Table 1

Table S1: Summary of sensitivity obtained in published papers.

| **Compound** | **Linear range (nM)** | **Source** |
| --- | --- | --- |
| SN 38 | 1.3 - 2550 | Khan, S. et al. A simple and sensitive LC/MS/MS assay for 7-ethyl-10-hydroxycamptothecin (SN-38) in mouse plasma and tissues: application to pharmacokinetic study of liposome entrapped SN-38 (LE-SN38). J Pharm Biomed Anal 37, 135–142 (2005). |
| MTX | 2.2 - 5500 | Michael S. Roberts et al. Determination of Methotrexate, 7-Hydroxymethotrexate, and 2,4-Diamino-N10-methylpteroic Acid by LC-MS/MS in Plasma and Cerebrospinal Fluid and Application in a Pharmacokinetic Analysis of High-Dose Methotrexate. Journal of Liquid Chromatography & Related Technologies, 39:16, 745-751 (2017) |
| DXd | N.A | No published analytical method in literature |
| MMAE | 1.4 - 3064 | Lee, B. ill et al. Liquid chromatography‐high resolution mass spectrometric method for the quantification of monomethyl auristatin E (MMAE) and its preclinical pharmacokinetics. Biomedical Chromatography 34, (2020). |
| MMAF | 4 - 3005 | Park et al. Pharmacokinetic and Metabolism Studies of Monomethyl Auristatin F via Liquid Chromatography-Quadrupole-Time-of-Flight Mass Spectrometry. Molecules 24, 2754 (2019). |
| CM | N.A | No published analytical method in literature |
